# Impact of advance care planning on dying in hospital: Evidence from urgent care records

**DOI:** 10.1371/journal.pone.0242914

**Published:** 2020-12-09

**Authors:** Martina Orlovic, Tom Callender, Julia Riley, Ara Darzi, Joanne Droney

**Affiliations:** 1 The Royal Marsden NHS Foundation Trust, London, United Kingdom; 2 Institute for Global Health Innovation, Imperial College London, London, United Kingdom; 3 Department of Applied Health Research, University College London, London, United Kingdom; University of Auckland, NEW ZEALAND

## Abstract

Place of death is an important outcome of end-of-life care. Many people do not have the opportunity to express their wishes and die in their preferred place of death. Advance care planning (ACP) involves discussion, decisions and documentation about how an individual contemplates their future death. Recording end-of-life preferences gives patients a sense of control over their future. Coordinate My Care (CMC) is London’s largest electronic palliative care register designed to provide effective ACP, with information being shared with urgent care providers. The aim of this study is to explore determinants of dying in hospital. Understanding advance plans and their outcomes can help in understanding the potential effects that implementation of electronic palliative care registers can have on the end-of-life care provided. Retrospective observational cohort analysis included 21,231 individuals aged 18 or older with a Coordinate My Care plan who had died between March 2011 and July 2019 with recorded place of death. Logistic regression was used to explore demographic and end-of-life preference factors associated with hospital deaths. 22% of individuals died in hospital and 73% have achieved preferred place of death. Demographic characteristics and end-of-life preferences have impact on dying in hospital, with the latter having the strongest influence. The likelihood of in-hospital death is substantially higher in patients without documented preferred place of death (OR = 1.43, 95% CI 1.26–1.62, p<0.001), in those who prefer to die in hospital (OR = 2.30, 95% CI 1.60–3.30, p<0.001) and who prefer to be cared in hospital (OR = 2.77, 95% CI 1.94–3.96, p<0.001). “Not for resuscitation” individuals (OR = 0.43, 95% CI 0.37–0.50, p<0.001) and who preferred symptomatic treatment (OR = 0.36, 95% CI 0.33–0.40, p<0.001) had a lower likelihood of in-hospital death. Effective advance care planning is necessary for improved end-of-life outcomes and should be included in routine clinical care. Electronic palliative care registers could empower patients by embedding patients’ wishes and personal circumstances in their care plans that are accessible by urgent care providers.

## Introduction

Place of death is an important outcome of end-of-life care, with implications for patients, caregivers and the healthcare system. Death in hospital is rarely a favourable outcome. More than half of individuals declare that they would prefer to be cared for and die at home or in a hospice [1–3], a preference that remains largely consistent throughout the course of disease [4, 5]. In spite of this, a substantial proportion of deaths still occurs in hospital [6]. In the United Kingdom (UK) almost every second death occurs in hospitals, while in the United States (US) it is every third [7, 8].

Over the last few decades, end-of-life care has become an increasingly important topic on the health policy agenda [3, 9]. There is a growing recognition that end-of-life care should be more patient-centric, and should aim to resolve the disconnect between patients’ end-of-life preferences for dying out-of-hospital and the care they receive in their last moments of life. Not knowing patient care preferences may lead to excessive utilisation of acute care and increased in-hospital deaths [10]. For many developed nations the cost of hospital care at end-of-life is significant [11, 12], increasing the pressure on national health budgets. However, transforming and improving such care is challenging due to its sensitive nature and the complex structure of health and social care systems.

Advance care planning (ACP) can support the provision of patient-centred and cost-effective care [13]. It involves discussion, decisions and documentation about how an individual contemplates their future death. Recording preferences about place of death and resuscitation decisions are important elements of advance planning, giving patients a sense of control over their future [14]. Having a recorded preferred place of death and a documented “not for resuscitation” order has been associated with a 76% greater chance of an individual dying where they wanted to die [15]. However, a significant proportion of dying patients do not have their preferred place of death documented [16].

Even though recording preferences is important, it is not a sufficient condition to ensure that these preferences are honoured [17]. A study indicated that for 53% of patients who recorded their terminal preferences, these were not found in their personal clinical records [18]. To support linking patient preferences to their care plans, electronic palliative care coordination systems (EPACCS), electronic registers designed to provide up-to-date information about patient care preferences, shared with relevant care providers including patients themselves, have been created [19]. Many countries have created their own electronic care registers, but emphasis on care coordination between different settings seems unique to the UK [19].

Coordinate My Care (CMC) is the largest EPACCS in England and serves as a clinical urgent care service provided by the National Health Service for London [20, 21]. Since its launch in 2010, care plans for over 83,000 individuals with incurable chronic or advanced disease have been recorded with this service [22]. CMC is designed to facilitate patients and their clinicians to record and make advance care decisions, before sharing them in real time with relevant healthcare professionals–including primary and secondary care providers, hospices, community and ambulance services–legitimately involved in patient’s care. Data collected as part of a CMC record includes demographics (age, gender, living circumstances, ethnicity, religion), clinical data (diagnoses, medications), patient preferences about end-of-life care (ceiling of treatment, preferred place of death and care, resuscitation status) and caregiver’s circumstances. Data is entered onto CMC plans by health care professionals in consultation with the individual patient and their family/carers. Patients also have the option to start creating a CMC plan themselves and enter their own personal data and information related to care preferences and wishes. This is then submitted digitally for their doctor or nurse to add the clinical data, confirm the plan with the patient and make it available on the CMC system. EPACCS such as CMC, can aid in recording, updating and disseminating these preferences with care providers to ensure these preferences are followed. There is a need for implementation of systems that enable linkage of advance records to patient’s care plans [23], and EPACCS is an example of that. Despite their potential, there is a lack of knowledge on how such EPACCS impact end-of-life outcomes [19].

Dying in one’s place of preference is often considered a quality indicator of end-of-life care [3]. Actual place of death can be influenced by a number of individuals characteristics and circumstances, for example underlying disease [5], health status [15], socio-economic status [5], ethnicity [24], as well as environmental factors such as the characteristics of health and social care system [6] and the availability of informal and formal care options [25]. Many of these factors are outside the control of the individual patient. By contrast, patients’ preferences regarding care are intrinsic to patients themselves. It has been shown that those with advance directives have higher likelihood of dying outside of hospital [26], although little is known about the impact that different end-of-life preferences have on that trajectory.

The identification of clinically relevant and modifiable factors, associated with increasing likelihood of dying outside of hospital is required to design sustainable health services that meet the wishes of terminally ill individuals in the context of growing ageing population [27, 28]. To address these gaps and expand the knowledge of EPACCS, CMC data have been used to explore determinants of dying in hospital, by focusing on patient characteristics and preferences regarding end-of-life care. Having insight into the content of patients’ advance plans, it is possible to examine the impact of different care preferences on the place of death which can have implications for health service strategic planning. Finally, investigating advance plans and their outcomes can help in understanding implications of EPACCS on the end-of-life care provided.

## Materials and methods

We included all individuals aged 18 or older with a CMC plan who had died between March 2011 and July 2019 and who had a recorded place of death. Information in the CMC plan was the latest available before individual’s death.

Analysis was performed using Stata (Version 14). Logistic regression was used to explore demographic and end-of-life preference factors associated with hospital deaths. The outcome variable was *“Dying in hospital”* where reference category included dying at “*Home”*, *“Care Home”*, “*Hospice*” and *“Other”* place of death. The model controls for gender, age, World Health Organisation (WHO) performance status, primary diagnosis, preferred place of death, preferred place of care, ceiling of treatment, resuscitation status and controls for the year of CMC record creation to control for time trends and controls for patient’s London geographic area to control for area variation. A “ceiling of treatment” is the documented decision about the level of clinical intervention that is deemed appropriate by medical professionals for an individual patient based on their previous and current medical needs and is often incorporated into local Treatment Escalation Plans [29].

As the non-recording of preferred place of death, ceiling of treatment and preferred place of care were likely to impact on place of death [15], we included this category in our analysis rather than imputing the data. The proportion of missing data for all other variables included in this analysis was 3% or less. CMC also collects a range of personal demographics variables, such as employment, living circumstances, ethnicity and religion, but because patients are reluctant to disclose this information, these variables had a large proportion of missing data (>60%) and therefore could not be included in the analysis. The study was approved by The Royal Marsden Hospital Committee for Clinical Research (Service Evaluation 860) and CMC is hosted by The Royal Marsden Hospital.

## Results

A total of 21,231 anonymised individual CMC records with recorded place of death were included. Descriptive results reveal significant variation in the actual place of death (Table 1). Only 22% of individuals died in hospital with 78% of patients dying in a non-hospital setting. Home (36%) and care home (27%) were the most common places of death. More than half of the cohort were 80 years of age or more (58%) and 54% were females. Just under half (49%) had a non-cancer terminal illness and 83% had a significantly restricted level of functioning (WHO performance status 3–4). When it comes to preferences regarding end-of-life care, for those who had the information available, most prefer to be cared at and die at home (61% and 44% respectively)rather than in a hospital, hospice or care home. A “not for resuscitation” decision had been made for most patients (79%). For nearly half, the ceiling of treatment was defined as for the treatment of symptoms only with the aim of comfort care (48%).

**Table 1:** Sample characteristics.

	Full cohort (N = 21,212)	Died out of Hospital* (N = 16,586)	Died in Hospital* (N = 4,626)
**Place of death**			
Hospital	4,626 (22%)	-	-
Home	7,709 (36%)	-	-
Hospice	3,147 (15%)	-	-
Care Home	5,645 (27%)	-	-
Other	85 (0%)	-	-
Not recorded	0 (0%)	-	-
***Age Group***			
<60	1,945 (9%)	1,497 (9%)	466 (10%)
60–69	2,459 (12%)	1,878 (11%)	581 (13%)
70–79	4,408 (21%)	3,427 (21%)	981 (21%)
≥80	12,400 (58%)	9,802 (59%)	2,598 (56%)
Not recorded	0 (0%)	-	-
**Gender**			
Male	9,678 (46%)	7,369 (44%)	2,309 (50%)
Female	11,534 (54%)	9,217 (56%)	2,317 (50%)
Not recorded	0 (0%)	-	-
**Diagnosis**			
Cancer	10,727 (51%)	8,619 (52%)	2,108 (46%)
Dementia	3,698 (17%)	3,147 (19%)	563 (12%)
Heart Disease	1,823 (9%)	1,261 (8%)	562 (12%)
Respiratory Disease	1,212 (6%)	841 (5%)	371 (8%)
Other	3,640 (17%)	2,700 (16%)	927 (20%)
Not recorded	23 (0%)	-	-
**WHO Performance Status**			
0 (Able to carry out all normal activity without restriction) & 1 (Restricted in strenuous activity but ambulatory and able to carry out light work)	769 (4%)	477 (3%)	292 (6%)
2 (Ambulatory and capable of all self-care but unable to carry out any work activities)	2,432 (11%)	1,594 (10%)	838 (18%)
3 (Symptomatic and in a chair or in bed for greater than 50% of the day but not bedridden)	6,061 (29%)	4,371 (27%)	1,690 (37%)
4 (Completely disabled; cannot carry out any self-care; totally confined to bed or chair)	11,418 (54%)	9,701 (60%)	1,717 (38%)
Not recorded	532 (3%)	-	-
**Preferred place of death**			
Care Home	5,103 (24%)	4,432 (27%)	671 (15%)
Home	9,328 (44%)	7,544 (45%)	1,784 (39%)
Hospice	1,716 (8%)	1,408 (8%)	308 (7%)
Hospital	210 (1%)	90 (1%)	120 (3%)
Other	236 (1%)	172 (1%)	64 (1%)
Not Recorded	4,619 (22%)	2,940 (18%)	1,679 (36%)
**Preferred place of care**			
Care Home	5,479 (26%)	4,721 (28%)	758 (16%)
Home	12,917 (61%)	10,072 (61%)	2,845 (62%)
Hospice	273 (1%)	222 (1%)	51 (1%)
Hospital	228 (1%)	86 (1%)	142 (3%)
Other	127 (1%)	98 (1%)	29 (1%)
Not Recorded	2,188 (10%)	1, 387 (8%)	801 (17%)
**Resuscitation Status**			
For resuscitation	1,124 (5%)	527 (3%)	597 (13%)
Not for resuscitation	16,693 (79%)	13,920 (85%)	2,773 (61%)
Not recorded	3,395 (16%)	2,139 (12%)	1,256 (26%)
**Preferred Place of Death Achieved**			
Yes	12,171 (57%)	11,885 (87%)	286 (10%)
No	4,422 (21%)	1,761 (13%)	2,661 (90%)
Not Recorded	4,619 (22%)	-	-
**Ceiling of Treatment**			
Treatment of any reversible conditions (including acute hospital)	6,122 (29%)	3,895 (29%)	2,227 (61%)
Symptomatic treatment only with the goal of keep comfortable	10,201 (48%)	9,076 (67%)	1,125 (31%)
Other	962 (5%)	648 (5%)	314 (9%)
Not recorded	3,927 (19%)	-	-
**Area**			
North Central London	1,685 (8%)	1,370 (8%)	315 (7%)
North East London	1,443 (7%)	970 (6%)	473 (10%)
North West London	5,264 (25%)	4,251 (26%)	1,013 (22%)
South East London	6,158 (29%)	4,899 (30%)	1,259 (27%)
South West London	6,502 (31%)	4,959 (30%)	1,543 (33%)
Other	160 (1%)	137 (1%)	23 (0%)
Not recorded	0 (0%)	-	-

Notes: Abbreviations: WHO—World Health Organisation; CCG—Clinical Commissioning Group

* missing data are excluded from % calculations.

**Fig 1:**
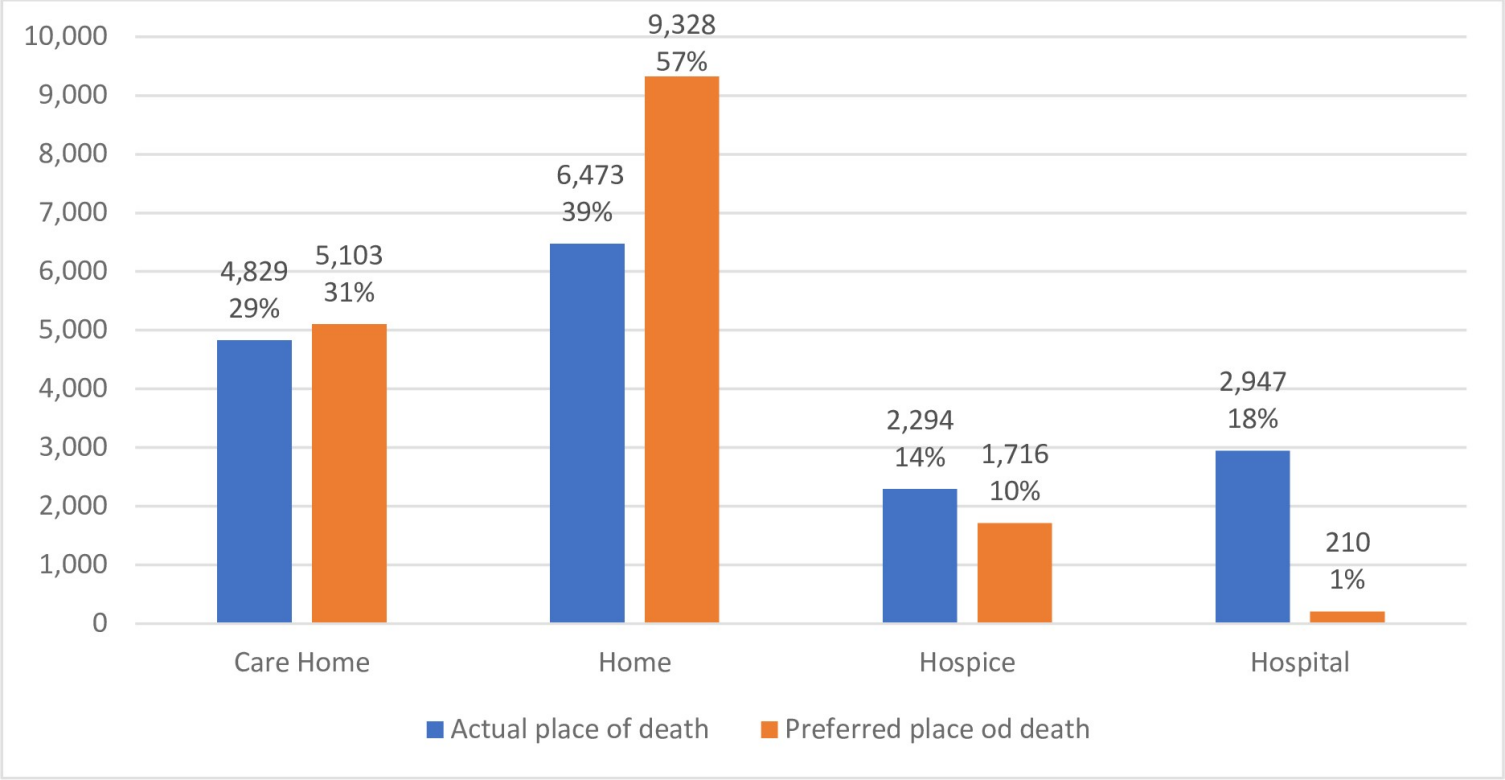
Comparison between actual and preferred place of death.

Overall, 73% of individuals for whom data on actual and preferred place of death is available, achieved their preferred place of death, although there are substantial differences between those who died in and outside of hospital. Excluding the individuals for whom the information is not available, 87% those dying outside of hospital achieved their preferred place of death as opposed to 10% who died in hospital. Only 210 (1%) of the study sample with a recorded preferred place of death chose to die in hospital, compared to 9,328 (56%) of those where home was their preferred place of death (Fig 1). Even though hospital was not the most common place of death, there is a stark difference between those who choose to die there and those who did die there.

A range of individual characteristics and preferences regarding end-of-life care are associated with dying in hospital (Table 2). Holding other variables constant, the odds of females dying in hospital rather than outside of hospital are 18% lower than for males. Being frail and disabled compared to those in relatively good health increases the likelihood of dying outside of hospital by 46%. When it comes to underlying diagnosis, having heart or respiratory disease substantially increases the chances of dying in hospital, compared to those who suffer from dementia ─ by 71% and 48% respectively. However, having cancer decreases the likelihood of dying in hospital by 27% compared to those with dementia.

**Table 2:** Logistic regression analysis of the probability of dying in a hospital.

Dependant variable–Dying in hospital		
(N = 17,203)		
Independent variable	OR (95% CI)	P value
**Gender (Ref = Male)**		
Female	0.82 (0.75–0.89)	*<0*.*001*
**Age> = 80 (Ref = Age<80)**	1.07 (0.98–1.17)	*0*.*152*
**WHO performance status (Ref = Level 0&1 merged)**		* *
2	1.02 (0.80–1.31)	*0*.*695*
3	0.95 (0.75–1.19)	*0*.*845*
4	0.54 (0.43–0.68)	*<0*.*001*
**Diagnosis (Ref = Dementia)**		* *
Cancer	0.73 (0.64–0.83)	*<0*.*001*
Heart Disease	1.71 (1.46–2.00)	*<0*.*001*
Respiratory Disease	1.48 (1.24–1.78)	*<0*.*001*
Other	1.48 (1.29–1.70)	*<0*.*001*
**Preferred place of death (Ref = Place of death specified but not hospital)**		* *
Hospital	2.30 (1.60–3.30)	*<0*.*001*
Not Recorded	1.43 (1.26–1.62)	*<0*.*001*
**Preferred place of care (Ref = Place of care specified but not hospital)**		* *
Hospital	2.77 (1.94–3.96)	*<0*.*001*
Not Recorded	1.09 (0.91–1.29)	*0*.*359*
Resuscitation Status (Ref = For resuscitation)*		* *
Not for resuscitation	0.43 (0.37–0.50)	*<0*.*001*
**Treatment ceiling (Ref = Full treatment)**		* *
Symptomatic treatment	0.36 (0.33–0.40)	*<0*.*001*
Other than above	0.68 (0.60–0.78)	*<0*.*001*

Notes: Results presented are from logistic regression analysis. Results are presented as odds ratios, indicating percentage odds change for a unit increase in the observed variable, holding other variables constant. N denotes sample size. OR denotes odds ratio. For dichotomous variables, reference group is the complementary category. Controls for Clinical Commissioning Area and year of enrolment are also included in each equation but suppressed from results table.

* Patients who do not have resuscitation status recorded are included in “For resuscitation” group as this is a default treatment strategy.

Compared to other observed characteristics, components of advance care planning have the strongest impact on the place of death. The likelihood of dying in hospital is substantially higher in patients who do not have a documented preference for the place of death (OR 1.43, 95% CI 1.26–1.62, p<0.001). If the preferred place of death is hospital, the likelihood of dying in hospital is more than doubled compared to individuals who preferred some other place of death (OR 2.30, 95% CI 1.60–3.30, p<0.001). Also, having hospital as their preferred place of care substantially increases the likelihood of dying in hospital (OR 2.77, 95% CI 1.94–3.96, p<0.001). Individuals who were “not for resuscitation” had a 57% lower chance of dying in hospital compared to those who were “for resuscitation”. Similarly, those who preferred symptomatic treatment were 64% less likely to die in hospital compared to individuals who preferred full treatment with more interventionist approach. Full statistical output is presented in S1 Table.

## Discussion

To the best of the authors’ knowledge, this is the largest EPACCS clinical service cohort analysis that examines factors associated with in-hospital death. CMC is a digital and multidisciplinary editable care platform, accessible via urgent care services enabling insight into patients’ contemporaneous end-of-life preferences. Analysis uses routinely collected data from CMC care plans that are created in partnership with clinical teams, patients and their family members, reflecting the CMC’s patient-centred approach. This contributes to the low proportion of missing data related to end-of-life care preferences and ensures that the information recorded is correct and up to date, which strengthens the analysis. Also, the CMC data are linked to the NHS Spine, a digital NHS platform used to exchange information in the system, which ensures that mortality is timely and correctly recorded.

Findings demonstrate the importance of individual characteristics and end-of-life preferences on dying in hospital. Health status is an important determinant of in-hospital deaths. Frail individuals and those suffering from long-term diseases such as cancer have a higher likelihood of dying out of hospital, contrary to individuals suffering from heart or respiratory disease. Furthermore, end-of-life preferences also play an important role when it comes to place of death. More specifically, information on preferred place of care and death, resuscitation status and ceiling of treatment are all patient’s end-of-life preferences that appear to have a profound impact on patient’s care. For most people, their aim in recording their end-of-life preferences is to relieve pain and prevent overtreatment at the end-of-life [30]. This was also observed for the CMC cohort as only 5% opted to be resuscitated, 29% wanted full treatment and only 1% have selected hospital as their preferred place of death. This information is only useful if recorded and accessible, as ACP is least likely to occur in hospitals, probably due to patient’s physical and mental state and a tendency to provide intensive treatments [31]. Giving patients an opportunity to communicate, update and record their end-of-life preferences is a step empowering patients and reducing complexity of decision-making in end-of-life care.

Dying in the preferred place of death is an indicator of end-of-life care quality [32, 33]. According to the latest National Survey of Bereaved People (VOICES) in England, 97% preferred to die outside of a hospital and 81% preferred to die in their usual place of residence [34]. Even though majority prefers to die outside of hospital, in 2018 46% of all deaths in England occurred in hospital, while the same proportion for London was 53% [7]. Over the past decades, in the US there has been an increase in the out-of-hospital deaths, due to inclusion of hospice care in Medicare’s benefits package [35, 36] and recognition of patient’s end-of-life preferences for non-acute terminal care [10]. In 2017, home became the most common place of death (30.7%), followed by hospital (29.8%) [8]. Among other things, further improvements are hindered by overutilization of high-intensity services at the end-of-life [10] and lack of an effective ACP process [17]. Even though home deaths increased, some evidence suggests that time in intensive care and care transitions in the last 3 months before death increased [37]. Due to hospital payment structures it is more financially beneficial if patients are kept in hospital on short term, so some may be inappropriately discharged early to be cared for at home where family members may be unprepared for the task [8]. Timely approach to ACP and managing care across settings is necessary to ensure “good death” at patient’s place of preference. In Scotland, patients with a Key Information Summary (KIS), the Scottish version of electronic palliative care register, had higher chances of dying in the community [38]. Observing the CMC cohort, the proportion of in-hospital deaths is significantly lower (22%) than in general UK public, and for whom data is available 73% died in their preferred place of death, demonstrating the impact of ACP and quality of end-of-life care for people with the CMC record. Further, since CMC patients’ records are linked to urgent care providers, it ensures that appropriate care is accessible round-the-clock. CMC, as an example of EPACCS, provides an opportunity to record and communicate contemporaneous end-of-life preferences to align patients’ wishes with end-of-life care provided.

Oregon in the US is a great example of the use of electronic registries of Physician Orders for Life-Sustaining Treatment (POLST) that allow patients to document their preferences on the use of life sustaining treatment at the end-of-life [17]. Use of POLST resulted in high rates of out-of-hospital deaths and higher likelihood to be discharged home in the last months of life [17]. Despite that, policy makers have highlighted that full potential of the programme has not been achieved because treating physicians could not always obtain these forms [17]. Also, POLST is focused mainly on future life-sustaining treatments, not full aspect of end-of-life care. It is filled out by a clinician and is usually implemented as a one-off service. CMC goes beyond by recording a breadth of end-of-life preferences, patients’ medical summary, personal and caregivers’ circumstances. In CMC patients can always access their records and update their preferences, facilitating information sharing and more effective decision-making [20]. Having such systems could align interests between patients and physicians and facilitate more efficient decision-making.

This study has several limitations. The analysed population includes terminally ill individuals with an urgent care plan who reside in Greater London. ACP completion rates in the general UK population are only around 8% [39], which is even a lower proportion of individuals whose ACP is accessible to urgent care providers [40]. Also, urban settings–such as London–tend to offer better access to different palliative and end-of-life care facilities, which can significantly impact the place of death, especially the probability of dying outside of hospital [41]. Therefore, the results might not be applicable to general UK population. Furthermore, due to the nature of the dataset, the analysis does not take into account factors such as socio-economic status, living circumstances, ethnicity and religion, all which might influence place of death [6, 10, 25, 42]. Also, even though the analysis includes the latest available care preferences of terminally ill patients, these preferences can evolve and change as patients are approaching death. Advance care planning is a process rather than a single time decision and changes in preferences may be updated in CMC as required. Therefore we cannot control for time from advance planning to death and lack of concordance between documented decisions and terminal outcomes could partly be due to the changing preferences regarding terminal care as individuals approach to death. Additionally, even though the CMC is a large dataset, we do not have a control group of patients without a CMC record and therefore we cannot attribute the patient outcomes to having a CMC record. Rather these data support the association between documenting and sharing ACP and achieving desired end-of-life outcomes. Finally, the cross-sectional design of the study hinders the ability to make causal inferences.

## Conclusion

Transforming end-of-life care requires dedicated support from all parties involved in care provision and there is no one-fits-all approach. Attention should be given to initiatives that respect patient wishes and improve quality of care provided. ACP is a way towards improved end-of-life outcomes and should be included in routine clinical care, so that patients and clinicians have enough time to discuss its purpose, available options and care implications. In light of the current COVID– 19 pandemic which threatens to overwhelm healthcare systems, supporting health care professionals to have ACP discussions with patients with advanced illness is highly important. Having insight into modifiable factors that significantly impact on the likelihood of death in hospital could influence someone’s terminal trajectory and end-of-life outcomes. Preventing overtreatment and effective care coordination at the end-of-life contributes to the cost-effective use of available resources. Electronic palliative care registers can be used to empower patients and to ensure that patients’ wishes and personal circumstances are embedded in their care plans.

## Supporting information

S1 Table Logistic regression analysis of the probability of dying in a hospital–full statistical output. (DOCX)
