# Supplementary material for: Impact of advance care planning on dying in hospital: Evidence from urgent care records
Source: PLoS One. 2020 Dec 9;15(12):e0242914. doi: 10.1371/journal.pone.0242914 (PMC7725362; doi:10.1371/journal.pone.0242914)
Supplement: S1 Table — (DOCX) [file pone.0242914.s001.docx]

# **S1 Table: Logistic regression analysis of the probability of dying in a hospital – full statistical output**

Number of observations = 117203

LR chi2(29) = 2054

Prob > chi2 = 0.0000

Log likelihood = -7333.4239 Pseudo R2 = 0.1200

| **Dependant variable: Dying in Hospital** | **Odds Ratio** | **SE** | **z** | **P>\|z\|** | **95% CI**  **LB** | **95% CI**  **UB** |
| --- | --- | --- | --- | --- | --- | --- |
| **Gender (Ref= Male)** |  |  |  |  |  |  |
| Female | 0.82 | 0.03 | -4.82 | ***0.000*** | 0.75 | 0.89 |
| **Age>=80 (Ref=Age<80)** | 1.07 | 0.05 | 1.43 | ***0.152*** | 0.98 | 1.17 |
| **WHO performance status (Ref= Level 0&1 merged)** |  |  |  |  |  |  |
| 1 | 1.02 | 0.13 | 0.20 | ***0.844*** | 0.80 | 1.31 |
| 2 | 0.95 | 0.11 | -0.47 | ***0.642*** | 0.75 | 1.19 |
| 3 | 0.54 | 0.06 | -5.26 | ***0.000*** | 0.43 | 0.68 |
| **Diagnosis (Ref=Dementia)** |  |  |  |  |  |  |
| Cancer | 0.73 | 0.05 | -4.82 | ***0.000*** | 0.64 | 0.83 |
| Heart Disease | 1.71 | 0.14 | 6.77 | ***0.000*** | 1.46 | 2.00 |
| Respiratory Disease | 1.48 | 0.14 | 4.23 | ***0.000*** | 1.24 | 1.78 |
| Other | 1.48 | 0.10 | 5.65 | ***0.000*** | 1.29 | 1.70 |
| **Preferred place of death (Ref=Place of death specified but not hospital)** |  |  |  |  |  |  |
| Hospital | 2.30 | 0.42 | 4.53 | ***0.000*** | 1.60 | 3.30 |
| Not Recorded | 1.43 | 0.09 | 5.61 | ***0.000*** | 1.26 | 1.62 |
| **Preferred place of care (Ref=Place of care specified but not hospital)** |  |  |  |  |  |  |
| Hospital | 2.77 | 0.50 | 5.62 | ***0.000*** | 1.94 | 3.96 |
| Not Recorded | 1.09 | 0.10 | 0.92 | ***0.359*** | 0.91 | 1.29 |
| **Resuscitation Status (Ref= For resuscitation)** |  |  |  |  |  |  |
| Not for resuscitation | 0.43 | 0.03 | -11.49 | ***0.000*** | 0.37 | 0.50 |
| **Treatment ceiling (Ref= Full treatment)** |  |  |  |  |  |  |
| Symptomatic treatment | 0.36 | 0.02 | -20.15 | ***0.000*** | 0.33 | 0.40 |
| Other than above | 0.68 | 0.05 | -5.67 | ***0.000*** | 0.60 | 0.78 |
| **Area (Ref= North Central London)** |  |  |  |  |  |  |
| North East London | 1.39 | 0.14 | 3.27 | ***0.001*** | 1.14 | 1.69 |
| North West London | 0.97 | 0.08 | -0.37 | ***0.709*** | 0.82 | 1.14 |
| South East London | 0.79 | 0.07 | -2.67 | ***0.008*** | 0.67 | 0.94 |
| South West London | 1.08 | 0.09 | 0.94 | ***0.346*** | 0.92 | 1.27 |
| Other | 0.81 | 0.25 | -0.68 | ***0.498*** | 0.44 | 1.49 |
| **Year (Ref=2011)** |  |  |  |  |  |  |
| 2012 | 0.70 | 0.83 | -0.30 | ***0.765*** | 0.07 | 7.06 |
| 2013 | 1.19 | 1.36 | 0.15 | ***0.879*** | 0.13 | 11.17 |
| 2014 | 1.07 | 1.22 | 0.06 | ***0.954*** | 0.11 | 9.93 |
| 2015 | 1.48 | 1.68 | 0.35 | ***0.730*** | 0.16 | 13.72 |
| 2016 | 1.45 | 1.65 | 0.33 | ***0.741*** | 0.16 | 13.46 |
| 2017 | 1.56 | 1.77 | 0.39 | ***0.696*** | 0.17 | 14.43 |
| 2018 | 1.60 | 1.82 | 0.41 | ***0.678*** | 0.17 | 14.83 |
| 2019 | 1.40 | 1.59 | 0.30 | ***0.768*** | 0.15 | 13.00 |
| **Constant** | 0.81 | 0.93 | -0.18 | ***0.856*** | 0.09 | 7.66 |

Notes: Results presented are from logistic regression analysis. Results are presented as odds ratios, indicating percentage odds change for a unit increase in the observed variable, holding other variables constant. For dichotomous variables, reference group is the complementary category.
